# MRI and Active Surveillance for Prostate Cancer

**DOI:** 10.3390/diagnostics10080590

**Published:** 2020-08-14

**Authors:** Angelo Porreca, Michele Colicchia, Gian Maria Busetto, Matteo Ferro

**Affiliations:** 1 Department of Urology, Policlinico di Abano, 35031 Abano Terme (PD), Italy; angeloporreca@gmail.com; 2 Department of Urology, Sapienza University of Rome, 00185 Roma, Italy; gianmaria.busetto@uniroma.it; 3 Division of Urology, European Institute of Oncology, IRCCS, 20141 Milan, Italy; matteo.ferro@ieo.it

**Keywords:** active surveillance, ·prostate cancer, MRI, imaging

Since the introduction of Prostate-Specific Antigen (PSA) screening, prostate cancer mortality has decreased [1], but over-diagnosis and overtreatment of this neoplasm have increased. This suggests a need for better prostate cancer risk stratification and an improvement in the detection of clinically significant prostate cancer [2]. During the last years, prostate cancer management has been revolutionized through the advent of active surveillance (AS). AS has evolved rapidly to become the standard of care for low-risk disease and a cautious option for intermediate-risk disease, because definitive treatment of prostate cancer is associated with significant morbidity and a negative impact on Quality of life [3,4]. 

Concurrently, multiparametric prostate magnetic resonance imaging (MRI), which combines T2-weighted imaging with functional imaging techniques (diffusion-weighted imaging (DWI), dynamic contrast-enhanced imaging, and magnetic spectroscopy) has been developed, and subsequently standardized in the Prostate Imaging Reporting and Data System (PI-RADS) [5].

Initially, MRI had been established in prostate cancer staging and diagnosis after a negative prostate biopsy. Moreover, the use of upfront MRI for prostate cancer diagnosis is increasing, because it may decrease the number of biopsy procedures and reduce the detection of clinically insignificant cancer. However, in a biopsy-naive setting, the improvement in the detection of clinically significant prostate cancer is less notable than in a non-biopsy-naïve setting. The prevalence of more aggressive prostate cancer is higher among men with a new diagnosis of low-risk prostate cancer than in the screened general population. Because positive and negative predictive values are sensitive to disease prevalence, MRI might be more useful in an AS setting compared to screening in the general population. Because MRI helps to identify higher grade and stage prostate cancer, its inclusion in AS care protocols is rational and is incorporated into European prostate cancer guidelines [3].

To determine the most appropriate patients for AS, several strategies based on clinical criteria (e.g., stage, Gleason score or grade group, and PSA), risk calculators, or nomogram have been proposed. However, the selection criteria for AS are still limited by the lack of prospective randomized clinical trials. Current literature based on these criteria, established prior to widespread MRI use, showed satisfactory outcomes for AS [6]. Additionally, a recent large cohort study investigating the impact of MRI on AS showed that rates of discontinuation, mortality, and metastasis in MRI-based AS were comparable with those of standard AS. However, it should be underscored that the median follow-up time was only 58 months, and the PI-RADS score was not used as a reporting tool [7].

The majority of recent studies with long-term follow-up have been limited to samples of AS men with very-low- or low-risk features. However, some men with intermediate-risk features, such as low-volume secondary Gleason pattern 4 or a PSA level >10 ng/mL may also be candidates [6].

The ability of MRI to detect primarily high-grade prostate cancers improves risk stratification for men under AS. MRI may be useful for identifying men with under-sampled or anterior lesions found on a standard prostate biopsy [8]. Multiple mostly single-institution studies have found that the combination of MRI and targeted prostate biopsy can improve patient selection compared to standard prostate biopsy alone. This suggests that a pre-biopsy MRI and MRI-targeted biopsies must be performed when indicated. However, at confirmatory biopsy, systematic biopsies maintain substantial added value [9]. Moreover, an initial report of a recent randomized study on the impact of MRI on AS failed to determine the advantage of pre-biopsy imaging. However, after a two-year follow-up, pre-confirmatory biopsy MRI was associated with a lower rate of failure of AS and prostate cancer progression to Gleason Grade Group >2 [10].

MRI may also be used for ongoing monitoring during AS. Traditionally, the follow-up strategy has been based on serial digital rectal examination, PSA, and repeated biopsy. Prostate biopsies have a high burden on Quality of Life during AS, but to minimize the risk of missing high-grade cancer, clinicians had encouraged its use. However, the high negative predictive value of MRI may negate the need for early repeat biopsies and offer the possibility for tailored biopsy strategies, particularly among men with under-sampled or anterior lesions on diagnostic biopsy [11]. However, MRI use in AS is still relatively new, additional work to understand markers and which patients are at highest risk is necessary. For example, changes in tumor volume need to be studied to understand better how MRI can be applied in conjunction with PSA and targeted biopsies. Here we must stress that MRI is not a substitute for biopsies, but may allow for more judicial use of them, thus improving patient quality of life and efficiency of care. Lately, a DETECTIVE consensus meeting concluded that men eligible for AS biopsy do not need any confirmatory biopsy after combined systematic- and MRI-targeted biopsies. MRI should also trigger prostate biopsy during AS when there are radiological changes such as an increase in PI-RADS score, lesion volume, and/or radiological T stage [12].

Unfortunately, there is still excessive heterogeneity in the literature on AS and its use; hence it is not surprising that currently, it is not definitive which AS strategy is superior and how MRI may be used and incorporated to best support AS in clinical practice.

The role of MRI in the management of prostate cancer is rapidly expanding. To improve the diagnostic ability and generalizability of MRI, several solutions are under investigation: ultra-high field magnetic resonance imaging, oxygen sensitive MRI, artificial intelligence, and “radiomics” (i.e., a prediction of histopathology and genetic signatures using many features from MRI) [13,14,15].

In conclusion, MRI has shown promising applications in AS for patient selection and monitoring. There still challenges to overcome in using MRI in AS, including standardizing interpretation and overcoming the learning curve. It is critical to note that MRI cannot be used as a substitute for repeat prostate biopsies but rather in conjunction with them, nor should there be a shift directly to immediate treatment. The next steps for including MRI into the standard of care in AS must include an accurate definition of the impact of MRI on AS including cost, quality, and access to care. Additionally, using newly available tools, like MRI-obtained prostate cancer features (e.g., number of lesions, PI-RADS, extra prostatic extension, seminal vesicle involvement), and/or biomarkers [16], new nomograms can be developed, which will allow for individually tailoring best AS strategy among men with prostate cancer.
